# Transcriptional reprogramming of metabolic pathways in critically ill patients

**DOI:** 10.1186/s40635-016-0094-1

**Published:** 2016-07-07

**Authors:** Marek Nalos, Grant Parnell, Robert Robergs, David Booth, Anthony S. McLean, Benjamin M. Tang

**Affiliations:** Nepean Genomic Research Group, Department of Intensive Care Medicine, Nepean Clinical School, University of Sydney, Sydney, Australia; Centre for Immunology and Allergy Research, Westmead Millennium Institute, Sydney, Australia; Queensland University of Technology, Brisbane, Queensland Australia

**Keywords:** Gene expression, Critical illness, Metabolism, Oxidation-reduction

## Abstract

**Background:**

Critical illness causes a shift away from mitochondrial metabolism towards a greater dependence on glycolysis. This metabolic shift is thought to be associated with lactic acidosis, organ dysfunction and poor clinical outcomes. The current paradigm is that low oxygen supply causes regional hypoxia, which in turn drives such a metabolic shift. In this study, we evaluated whether the shift towards glycolysis can also occur in cells where oxygen supply is plentiful.

**Methods:**

We used circulating blood cells from non-hypoxic critically ill patients (*n* = 47) as a model to study cellular metabolism in a normal oxygen milieu. We measured the transcriptomic profiles of canonical metabolic pathways in these cells and compared them to cells obtained from healthy controls (*n* = 18).

**Results:**

Transcriptomic profiling revealed a significant reprogramming of metabolic pathways during critical illness. In well-oxygenated cells, there was a reduced expression of tricarboxylic acid cycle genes and genes associated with pyruvate entry into the mitochondria suggesting decreased mitochondrial oxidation. In contrast, glycolysis was accelerated, as reflected by an up-regulation of genes coding for enzymes of early and late glycolytic pathway that were associated with increased lactate production. The pentose phosphate pathway genes for NADPH production were also up-regulated suggesting enhanced antioxidant production during increased oxidative stress.

**Conclusions:**

Contrary to the established paradigm, aerobic glycolysis does occur in non-hypoxic cells during critical illness and its occurrence may represent an adaptive strategy common to cells under increased oxidative stress. Further study of this previously under-recognized metabolic phenomenon might identify novel drug target for antioxidant therapy.

**Electronic supplementary material:**

The online version of this article (doi:10.1186/s40635-016-0094-1) contains supplementary material, which is available to authorized users.

## Background

In health, energy production from carbohydrate substrates is predominantly driven by mitochondrial oxidation and to a lesser extent by glycolysis. In times of increased physiological stress, this mitochondrial/cytosol balance is reversed favouring glycolysis as the dominant form of energy production [1]. Critical illness represents the most severe form of physiological stress to the human host. Stressed leukocytes in septic patients, for example, shift from mitochondrial oxidation to an increased dependence on glycolysis to meet their extraordinarily high metabolic demand (e.g. respiratory burst or phagocytosis) [2]. The physiological trigger for this metabolic shift, however, is largely unknown.

Ischemia may cause the metabolic shift, based on the well-known observations that anaerobic metabolism was associated with an increased glycolysis [3]. However, this theory fails to account for many situations in which ischemia is not a prominent feature (e.g. sepsis). An alternative explanation is that systemic inflammation may trigger the glycolysis. This is based on studies showing that inflammation is associated with a significant metabolic shift in activated leukocytes [2]. In addition, emerging evidence showed that metabolic shift may actually represent an adaptive physiological response to systemic inflammation in critical illness, even when an ischemic stimulus is absent [4]. This leads us to hypothesize that a metabolic switch may occur in non-hypoxic conditions where ischemia is absent but systemic inflammation is a prominent feature, such as sepsis, trauma or surgery. To test our hypothesis, we conducted a longitudinal study in patients with sepsis or systemic inflammatory response syndrome (SIRS) to determine whether critical illness is associated with a metabolic shift in cells with normal oxygen supply. We chose sepsis and SIRS patients because systemic inflammatory response was a common feature in these patients. We choose circulating leukocytes as our target tissue because these cells were bathed in oxygenated blood with plentiful supply of oxygen, hence representing an ideal environment to study metabolic changes in a normal/high oxygen milieu.

## Method

### Subjects

The study had a total of 65 subjects, including (1) 35 patients with sepsis, (2) 12 patients with SIRS and (3) 18 healthy volunteers as controls. The study protocol and its raw data have been previously published elsewhere [5]. The study was approved by the Sydney West Area Health Service Human Research Ethics Committee. Informed written consent was obtained from all patients or their relative and from healthy volunteers.

Systemic inflammatory response syndrome (SIRS) was defined as the presence of at least two of the four clinical criteria: (a) fever or hypothermia (temperature >38 °C [100.4 F] or <36 °C [96.8]); (b) tachycardia (>90 beats/min); (c) tachypnea (>20 breaths/min or PaCO_2_ < 32 mmHg [4.3 kPa]) or the need for mechanical ventilation; (d) an altered white blood cell count of more than 12,000 cells/L, less than 4000 cells/L, or the presence of more than 10 % band forms. Sepsis was defined as bacterial infection in addition to the presence of at least two of the four criteria above. The sepsis inclusion criteria required confirmation by microbiological culture and consensus by the consulting physician that sepsis was the cause of the patient’s presenting illness. The consulting physician was blind to the outcome measures (see below) of the study.

### Samples

We collected whole blood samples from each study participant within 24 h of admission to the intensive care unit, henceforth referred to as day 1. Daily samples collection was performed for up to 5 days. Sample collection in healthy volunteers was performed only on days 1 and 5.

### RNA extraction and microarray experiments

RNA extraction was performed using the standard protocol in batches of 12 to 24 samples at a time (PAXgene Blood RNA kit; Qiagen, Germany). RNA quality was analysed using the Agilent 2100 Bioanalyser (Agilent Technologies, Santa Clara, CA), and all samples obtained an RNA integrity number of greater than 6.5, indicating high sample integrity. Extracted RNA was stored at −80 °C until expression profiling using Illumina Sentrix HT-12_v3_BeadChip arrays (Illumina, San Diego, California). Sample amplification and labelling were carried out on 200 ng of total RNA using an Illumina TotalPrep Amplification kit (Ambion, Austin, Texas) in batches of 24 samples at a time. Amplified cRNA was assessed using the 2100 Bioanalyser to ensure satisfactory amplification. The samples were then immediately hybridized onto HT-12_v3_BeadChips. Each sample (750 ng) was loaded onto the arrays. The hybridization and washing procedure were identical for each set of arrays processed and, after normalization, no significant batch effects were identified. To minimize experimental artefacts, all of the RNA extraction, sample amplification and labelling, hybridization and washing and scanning procedures were carried out by the same operator at the same time of the day.

Transcriptomic raw data were obtained by scanning of the microarray slides using Illumina GenomeStudio V2010.3. Each probe on the array was passed through a filter requiring a detection *p* value of <0.0050 in at least one sample to be included in any further analyses. Of the 48,804 probes present on the Illumina HT 12 array, 24,840 probes (henceforth referred to as genes) passed this criterion. Genes that passed the filtering were loaded into BRB ArrayTools, in which quantile normalization and log transformation of the data were applied.

### Gene-expression experiments

We used transcriptomic profiling to study the metabolic pathways since previous studies had shown that cellular metabolism was regulated at a transcriptional level [4, 6, 7]. Blood samples were collected into PAXgene tubes (Pre-Analytix, Switzerland) and were subsequently processed for RNA extraction using the manufacturer’s protocol (PAXgene Blood RNA kit; Qiagen, Germany). Extracted RNA was then used in microarray experiments using Illumina Sentrix HT-12_v3_BeadChip arrays (Illumina, San Diego, California). Raw data were obtained by scanning of the microarray slides using Illumina GenomeStudio V2010.3. Of the 48,804 probes present on the Illumina HT 12 array, 24,840 probes (henceforth referred to as genes) passed quality criterion. Additional information on the microarray experiments and the statistical methods used to analyse the microarray data is provided in the Additional files 1, 2 and 3. Raw microarray data of the entire data set is available in GEO (GSE54514).

### Pathway analysis

**Fig. 1:**
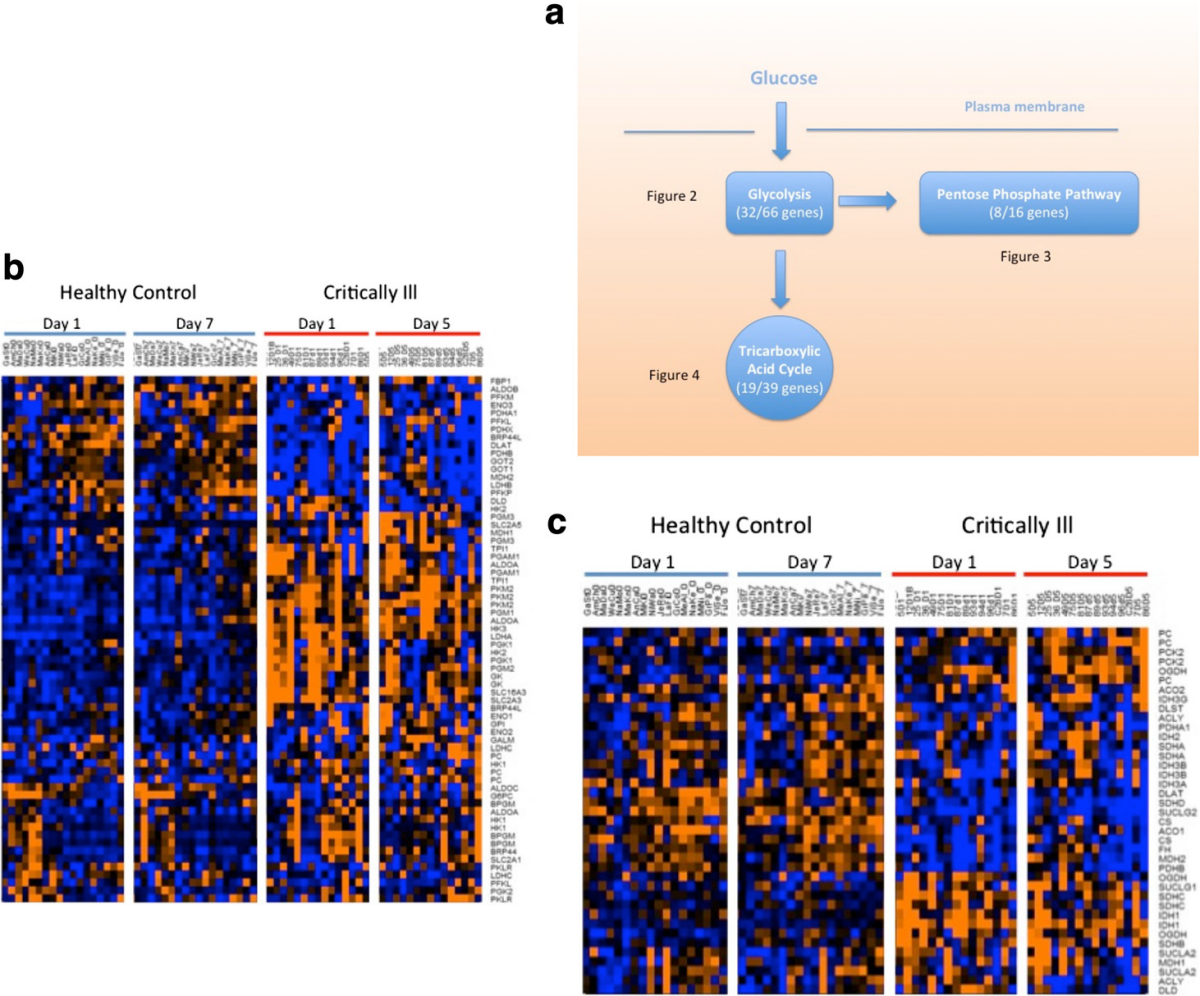
**a** Metabolic pathways of circulating leukocytes from non-hypoxic critically ill patients. *Data inside the parentheses* indicates the total number of genes (*denominator*) included in pathway analysis and the number of genes (*numerator*) that were statistically significantly different between critically ill patients and healthy controls. **b** Heat map of the glycolysis pathway. Each *row* represents a gene, and each *column* represents a sample. *Red colour* denotes up-regulation, and *blue colour* denotes down-regulation. For critically ill patients, the study period was 5 days. For healthy volunteers, the study period was 7 days. **c** Heat map of the tricarboxylic acid pathway. Each *row* represents a gene, and each *column* represents a sample. *Red colour* denotes up-regulation and *blue colour* denotes down-regulation. For critically ill patients, the study period was 5 days. For healthy volunteers, the study period was 7 days

We focused our analysis on genes involved in the canonical metabolic pathways of glycolysis, tricarboxylic acid cycle and pentose phosphate pathway. We performed a student’s *t* test in each gene to compare the expression levels between the healthy controls and critically ill patients. Differentially expressed genes that were identified to be statistically significant were then visualized using PathVisio 3.2.1 and wikipathways (WP534_78585_glycolysis; WP78_70014_TCA; WP134_68931_ppp; http://wikipathways.org, accessed 05-05-201). The number of genes in each pathway is shown in Fig. 1.

### Statistical analysis

We focused our analysis on genes involved in the canonical metabolic pathways of glycolysis, tricarboxylic acid cycle and pentose phosphate pathway. For each gene involved in these pathways, we performed a *t* test to compare the expression level between the healthy controls and the critically ill patients diagnosed with sepsis or systemic inflammatory response syndrome. A threshold of *p* < 0.01 was used to determine significance of differences observed.

## Results

### Patient characteristics

A total of 47 study subjects (35 sepsis and 12 SIRS) and 18 healthy volunteers were enrolled into our study. The study subjects had a wide range of critical illnesses, including surgical and medical diseases involving several major organ systems (cardiovascular, pulmonary, metabolic, renal, hepatic systems) (Table 1). All study subjects fulfilled criteria for systemic inflammatory response syndrome. The mean arterial oxygen tension levels were 124 mmHg (normal range 60–100 mmHg), indicating that the circulating leukocytes were well oxygenated in most subjects. Furthermore, expression levels of hypoxic-inducible factor 1-alpha (HIF1-α) gene in our patient were not increased arguing against hypoxia (Additional file 2).

**Table 1:** Patient demographics and clinical characteristics

	Sepsis	SIRS	Healthy controls
Demographics			
Number in group	35	12	18
Mean age (year)	64	61	43
Severity of illness			
Mortality (%)	26	0	0
Mean APACHE II	20	16	NA
Vasopressor support (%)	72	16.8	0
Renal support (dialysis) (%)	26	8.3	0
Mechanical ventilation (%)	78	75	0
Presentation (*n*)^a^			
Pulmonary infection	17	0	0
Urinary tract infection	4	0	0
Abdominal sepsis	6	0	0
Septicaemia	3	0	0
Soft tissue infection	3	0	0
Infective endocarditis	2	0	0
Post emergency surgery	7	1	0
Trauma	1	1	0
Myocardial infarction	0	3	0
Severe pancreatitis	0	1	0
Acute pulmonary oedema	0	2	0
Acute haemorrhage	0	2	0
Liver failure	0	1	0
Cardiogenic shock	0	2	0
Arrhythmias	0	2	0
Cerebral infarction	0	2	0
Pre-existing illness (*n*)^a^			
Hypertension	9	7	3
Ischaemic heart disease	9	4	0
Chronic lung disease	8	2	0
Cancer	6	0	0
Diabetes	7	3	0

*SIRS* systemic inflammatory response syndrome, *APACHE* Acute Physiology and Chronic Health Evaluation

^a^Some patients have multiple conditions

### Overall findings

We found major changes in the metabolic pathways of the critically ill patients compared to the healthy controls. Nearly 50 % of the genes displayed changes during critical illness (Fig. 1a). Heat maps of the pathway genes revealed that these changes followed a distinctive pattern (Fig. 1b, c). In the resting state (i.e. healthy controls), the glycolytic pathway gene transcription was quiescent while that of tricarboxylic acid cycle was active. During critical illness, this pattern was reversed; the glycolytic genes became highly expressed while the previously active transcription of tricarboxylic acid cycle genes was down-regulated (Fig. 1b, c). These findings suggested that a major reprogramming had occurred in the metabolic pathways of the cells.

**Fig. 2:**
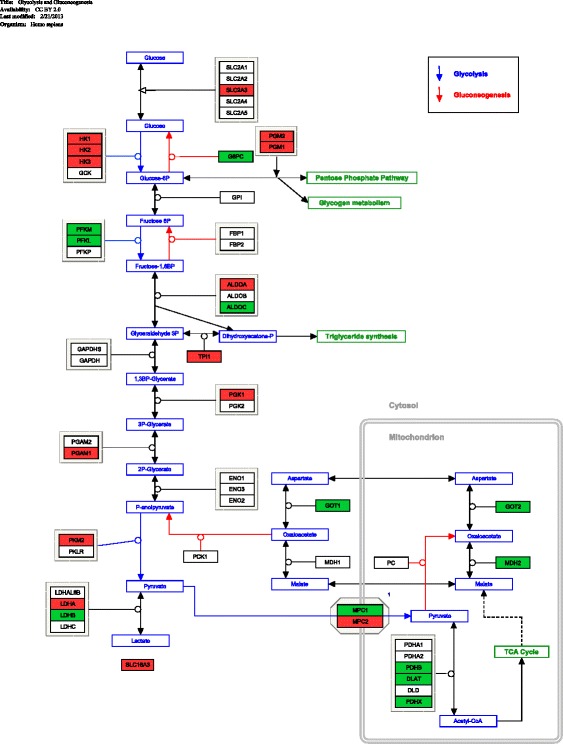
Pathway diagram of the glycolysis pathway. Statistically significant genes (*p* < 0.01) (between critically ill and healthy controls) are coloured in either *red* or *green. Red colour* denotes up-regulation, and *green colour* denotes down-regulation. *Lines with one-direction arrow* (i.e. irreversible reaction) represent key regulatory control steps of the pathway. *Lines with arrows on both directions* denote reversible reactions determined by mass effect (i.e. forward and backward reaction can occur depending on substrate concentration)

**Fig. 3:**
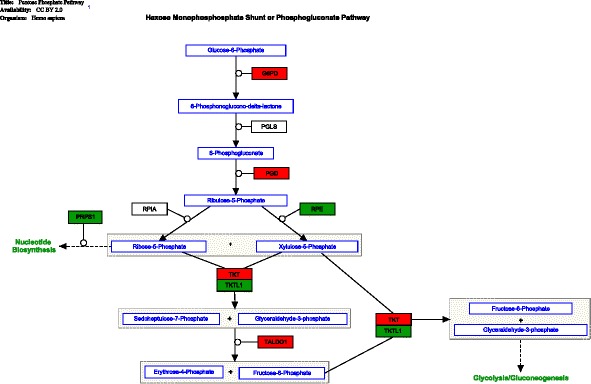
Pathway diagram of the genes in the pentose phosphate pathway. Statistically significant genes (*p* < 0.01) (between critically ill and healthy controls) are coloured in either *red* or *green. Red colour* denotes up-regulation, and *green colour* denotes down-regulation. *Lines with one-direction arrow* (i.e. irreversible reaction) represent key regulatory control steps of the pathway. *Lines with arrows on both directions* denote reversible reactions determined by mass effect (i.e. forward and backward reaction can occur depending on substrate concentration)

**Fig. 4:**
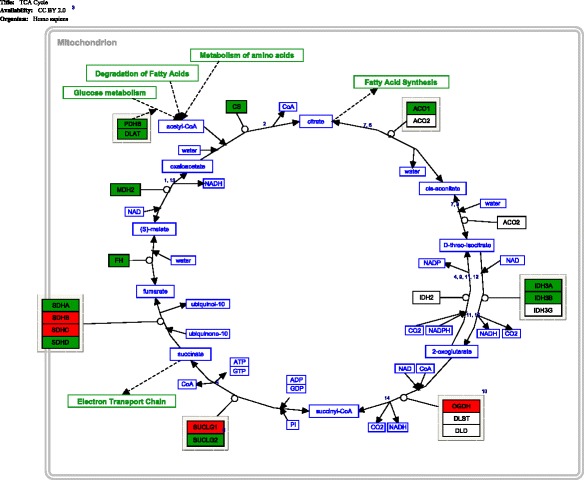
Pathway diagram of the genes in the tricarboxylic acid cycle. Statistically significant genes (*p* < 0.01) (between critically ill and healthy controls) are coloured in either *red* or *green. Red colour* denotes up-regulation, and *green colour* denotes down-regulation. *Lines with one-direction arrow* (i.e. irreversible reaction) represent key regulatory control steps of the pathway. *Lines with arrows* on both directions denote reversible reactions determined by mass effect (i.e. forward and backward reaction can occur depending on substrate concentration)

This metabolic reprogramming persisted throughout the study period (5 days). Importantly, it did not vary with the severity of the illness since similar changes were found in both SIRS and sepsis subgroups despite the fact that sepsis subgroup had higher APACHE scores (hence more severe disease) and a lower survival rate. Therefore, we present the data henceforth in reference to all patients (“critically ill patients”) unless otherwise specified. Details of these changes are given in Figs. 2, 3 and 4 (see below) and the full analysis are also provided in Additional files 1, 2 and 3.

### Major change (1)—glycolysis pathway

There are three main control points that regulate glycolysis. We found that genes for two of the three control points were up-regulated in the critically ill patients compared to the healthy controls (Table 2 and Fig. 2). The first was hexokinase (gene symbol: *HK*). This enzyme converts glucose to glucose-6-phosphate, the essential first step of glycolysis. We found gene-expression up-regulation in all three isoforms of this enzyme (*HK1*, *HK2*, *HK3*), with one isoform (i.e. *HK1*) displaying the highest increase in fold change (3.88–5.82) among all genes (Additional file 1: Table S1). The second gene was pyruvate kinase (*PKM2*) and its expression level was also increased in the critically ill patients compared to the healthy control. This enzyme controls the final step of glycolysis; it produces energy (ATP) and pyruvate as end products. The increased activity in these two main key control enzymes (i.e. hexokinase and pyruvate kinase) would suggest that an accelerated glycolysis had occurred in the critically ill patients. As further evidence, the glucose transporter gene *SLC2A3* was up-regulated. *SLC2A3* has fivefold higher transport capacity than other isoforms of the same gene (*SLC2A1*, *SLC2A2* and *SLC2A4).* The up-regulation of this gene suggested that the glucose transport across the cell membrane was at its maximal capacity, due to a high glucose demand generated by an accelerated glycolysis.

**Table 2:** Representative genes in glycolytic pathway

Symbols	Isoforms	Gene names	Functions	Roles in glycolysis	Our findings
*SLC2A*	*SLC2A1*, *SLC2A2*, *SLC2A3*, *SLC2A4*, *SLC2A5*	Solute carrier family 2 facilitated glucose transporter	Transport glucose molecules across the cell membrane	Provides glucose for glycolytic pathway	Up-regulation
*HK*	*HK1*, *HK2*, *HK3*	Hexokinase	Initiate glycolysis by conversion of glucose to glucose-6-phosphate	The first essential step in glycolysis	Up-regulation
*G6P*	*G6PC*	Glucose-6-phosphate catalytic subunit	Hydrolyse glucose-6-phosphate to glucose	Reverse glycolysis	Down-regulation
*PK*	*PKM2*, *PKLR*	Pyruvate kinase	Generates ATP and pyruvate as the final products	The final step in glycolysis	Up-regulation
*LDH*	*LDHA*	Lactate dehydrogenase	Conversion of pyruvate to lactate	Post-glycolysis removal of pyruvate	Up-regulation
	*LDHB*	Lactate dehydrogenase	Convert lactate back to pyruvate	Provide pyruvate for mitochondria oxidation	Down-regulation

We found additional evidence of increased glycolysis. In resting cells, *G6PC* provides an exit point for excess glucose to leave the glycolytic pathway. Here, we found that *G6PC* was significantly down-regulated, further corroborating that the condition was favourable for an increased glycolysis (Fig. 2). In addition to an increased glycolysis, we also observed gene-expression signals suggesting an increased downstream processing of the glycolysis end product, the pyruvate (Fig. 2). This downstream processing displayed two important features. First, the gene expression of *LDHA* was up-regulated and that of *LDHB* down-regulated suggesting increased forward reaction of lactate dehydrogenase (pyruvate→lactate) indicating that pyruvate was increasingly converted to lactate. Second, this phenomenon was accompanied by the up-regulation of *SLC16A3* gene, which codes for monocarboxylate transporter 4 that shuttles lactate out of the cell. In keeping with the above findings, we noted that gene-expression levels of the two key glycolytic enzymes *HK3* (first step in glycolysis) and *LDHA* (last step in glycolysis) correlated with increased serum lactate (Additional file 1).

In summary, we observed transcriptional changes suggesting that the glycolytic pathway has accelerated, as evidenced by (1) increased glucose transport into the cell, (2) up-regulation of key enzymes and (3) increased end product. Interestingly, these changes further suggest that pyruvate, the major end product, was converted into lactate and subsequently shuttled out of the cell, thereby bypassing the tricarboxylic acid cycle altogether. This was unexpected since traditional paradigm suggested that, when the conditions were favourable (i.e. plentiful oxygen supply), pyruvate would enter the tricarboxylic acid cycle for ATP synthesis; instead, we observed an increased transport of pyruvate out of the cell.

### Major change (2)—pentose phosphate pathway

We found evidence that metabolic intermediates generated by increased glycolysis were diverted to the pentose phosphate pathway (PPP). There were several findings to support this. First, phosphofructokinase gene (*PFK*), a key regulatory enzyme in the early glycolytic pathway, was down-regulated (including both isoforms *PFKM* and *PFKL*). This suggested a substantial shift towards the PPP (Fig. 2). Further evidence of a diverted glycolysis was provided by the genes *PGM1* and *PGM2*. Enzymes encoded by *PGM1* and *PGM2* normally up-regulate in response to excessive glucose-6-phosphate present in the cytosol. We found that their expression levels were increased, suggesting that acceleration towards the PPP had indeed occurred. More direct evidence of an accelerated pentose phosphate pathway was provided by up-regulation of genes coding for two key enzymes, glucose-6-phosphate dehydrogenase (*G6DP*) and phosphogluconate dehydrogenase (*PGD*). They are major enzymes in the early part of the pentose phosphate pathway (Table 3).

**Table 3:** Representative genes in pentose phosphate pathway

Symbols	Gene names	Functions	Roles in pentose phosphate pathway	Our findings
*G6PD*	Glucose-6-phosphate dehydrogenase	Produce NADPH	Rate-controlling enzyme that initiates the pathway	Up-regulation
*PGD*	Phosphogluconate dehydrogenase	Produce NADPH	Complete the oxidative phase of the pathway	Up-regulation
*PRPS1*	Phosphoribosyl pyrophosphate synthetase 1	Support biosynthesis of nucleotide	Non-oxidative phase of the pathway	Down-regulation
*RPE*	Ribulose-5-phosphate-3-epimerase	Support biosynthesis of nucleotide/amino acid and support glucose metabolism	Non-oxidative phase of the pathway	Down-regulation

The pentose phosphate pathway consists of two parts: the early part (the oxidative phase) is responsible for generating NADPH and the late part (the non-oxidative phase) for biosynthesis (nucleic acids, enzymes or co-factor) [8]. We found a preferential up-regulation of the early part over the late part (Table 3). Both NADPH-producing enzymes (*G6DP* and *PGD*) displayed high gene-expression levels (Additional file 1), indicating a substantially increased NADPH synthesis in these cells. There were two further pieces of evidence to support preferential increase in NADPH production over biosynthesis. First, the biosynthetic component of the pentose phosphate pathway was down-regulated (Fig. 3). Second, in a post hoc analysis, we examined regulatory genes that govern cell proliferation/growth (e.g. *Myc*, *ATK1*, *PIK3*, *HIF1*-α) [9]. This analysis showed that there was no evidence of up-regulation in these genes (Additional file 2), indicating that the cellular demand for biosynthesis was absent.

### Major change (3)—tricarboxylic acid cycle

We observed a down-regulation in genes that control the entry point into the tricarboxylic acid cycle (Fig. 4). This down-regulation occurred in all major genes of the pyruvate dehydrogenase complex, namely, dihydrolipoamide S-acetyltransferase (*DLAT*), component X (*PDHX*) and component beta (*PDHB*) (Table 4). There was additional evidence of inhibition in the tricarboxylic acid cycle. First, the mitochondrial pyruvate carrier gene (*MPC1, MPC2*) was not up-regulated, as would be expected in an environment of an increased glycolysis (which would have resulted in more pyruvate transport into the mitochondria). Second, malate dehydrogenase gene (*MDH2*) was also down-regulated. If confirmed on a protein level, this down-regulation may represent less oxaloacetate available to combine with acetyl-CoA, the essential first step in the tricarboxylic acid cycle. Third, isocitrate dehydrogenase genes (*IDH3A*, *IDH3B*) and malate dehydrogenase gene (*MDH2*) were down-regulated (Table 4). The enzymes coded by those genes produce NADH, the reducing potential carrier essential for oxidative phosphorylation in the mitochondria. In summary, we found transcriptomic evidence of inhibition in the tricarboxylic acid cycle in terms of substrate supply, pathway initiation and relay into mitochondrial oxidation. Together with an increased pentose phosphate pathway activity, these findings suggest that the cell was shifting its activity away from mitochondria (oxidative) and moving towards the pentose phosphate pathway (reductive).

**Table 4:** Representative genes in tricarboxylic acid cycle (TCA) pathway

Symbols	Isoforms	Gene names	Functions	Roles in TCA pathway	Our findings
*IDH*	*IDH3A*, *IDH3B*	Isocitrate dehydrogenase	Decarboxylation of isocitrate to 2-oxoglutarate	Generate NADH and carbon dioxide	Down-regulation
*MDH2*	*MDH2*	Malate dehydrogenase	Oxidation of malate to oxaloacetate	Generate NADH and carbon dioxide	Down-regulation
*PDHB*	*PDHB*	Pyruvate dehydrogenase component beta	Convert pyruvate to acetyl-CoA	Entry point into the TCA pathway	Down-regulation
*PDHX*	*PDHX*	Pyruvate dehydrogenase complex, component X	Convert pyruvate to acetyl-CoA.	Entry point into the TCA pathway	Down-regulation
*DLAT*	*DLAT*	Pyruvate dehydrogenase complex dihydrolipoamide S-acetyltransferase	Convert pyruvate to acetyl-CoA.	Entry point into the TCA pathway	Down-regulation

## Discussion

**Fig. 5:**
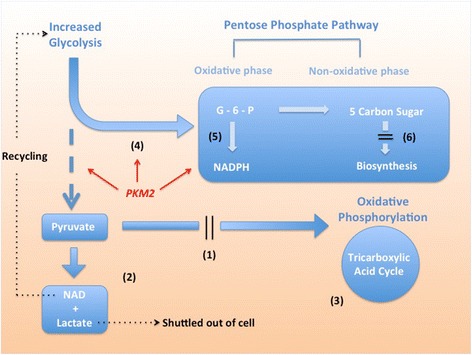
Summary of metabolic reprogramming and increased glycolysis in circulating leukocytes during critical illness. (*1*) Reduced entry into the oxidative phosphorylation. (*2*) diverting pyruvate away from oxidative phosphorylation. (*3*) Reduced activity in the tricarboxylic acid cycle. (*4*) Increased substrate supply to the pentose phosphate pathway. (*5*) Increased NADPH production. (*6*) Reduced biosynthesis. *PKM2* pyruvate kinase 2, *NADPH* nicotinamide adenine dinucleotide phosphate, *NADH* nicotinamide adenine dinucleotide *G-6-P* glucose-6-phosphate

Oxidative phosphorylation in the mitochondria is thought to be the most efficient pathway for energy production in aerobic conditions [10]. This high efficiency comes with a cost; oxidative phosphorylation produces reactive oxygen species as a by-product. The accumulation of reactive oxygen species (ROS) increases oxidative stress to the cell with deleterious effects on function and cell survival [11]. In healthy cells, the harmful effect of ROS is nullified by antioxidant NADPH (generated by pentose phosphate pathway), such that a stable redox homeostasis is maintained. In critical illness, pro-inflammatory cytokines (tumour necrosis factor-α, interleukin-1β) increase the amount of ROS dramatically, thereby tilting the redox balance towards an oxidative environment [12]. Here, in a study of patients with systemic inflammatory response syndrome and sepsis, we showed that circulating leukocytes might have adapted to this oxidative environment by a reprogramming of metabolic pathways. This metabolic reprogramming was suggested by gene-expression changes including (1) reduced substrate entry into oxidative phosphorylation, (2) diverting substrate pyruvate away from oxidative phosphorylation, (3) reduced activity of the tricarboxylic acid cycle, (4) increased substrate supply to the pentose phosphate pathway, (5) increased NADPH production in the pentose phosphate pathway and (6) reduced biosynthesis. The net outcome was an increase in transcriptional activity of enzymes required for glycolysis, lactate production and NADPH synthesis. A summary of these steps is given in Fig. 5.

Our data suggest that the driving force behind this reprogramming was an increased dependence on aerobic glycolysis. The glycolytic pathway provided not only substrates for energy production but also the enzymes that linked together key steps of this metabolic reprogramming. The most notable example was pyruvate kinase (gene symbol: *PKM2*), which participates in both glycolysis and redox homeostasis (Fig. 5). It is known that two isoforms of pyruvate kinase exist in normal cells (PKM1, PKM2) [13]. In this study, we found that the gene for the slower isoform *PKM2* was preferentially expressed in the critically ill patients. This isoform PKM2 has a slower enzyme kinetic such that its activation results in an accumulation of earlier glycolytic intermediates, which may then be diverted towards the pentose phosphate pathway [14]. In addition, PKM2 itself indirectly activates the pentose phosphate pathway, further facilitating the NADPH production [15]. Finally, recent studies have demonstrated the presence of a PKM2-ROS-pentose phosphate pathway feedback loop in human cells, further confirming the role of PKM2 up-regulation in redox balance [14]. Put together, these findings suggest that glycolytic enzymes are part of an integrated network of redox homeostasis. Our study is the first report to show that such a network may exist in critically ill patients.

While novel within the critical care context, our finding that glycolysis participates in metabolic reprogramming has been well observed in oncology [16]. Rapidly growing cancer cells are known to mobilize the glycolytic pathway in order to recruit the pentose phosphate pathway, which produces macromolecules (e.g. nucleic acid, protein, lipid) to support cancer growth and proliferation. Our findings differ from these oncological observations in one important aspect; that the recruitment of the pentose phosphate pathway was not for the purpose of biosynthesis but rather to increase NADPH production. In critical illness, an ample supply of NADPH helps regulate cell function, assists pathogen killing and protects against free radical damage.

Approximately 60 % of NADPH production in human is by the pentose phosphate pathway [8]. The pentose phosphate pathway is normally quiescent (only 1.3 % of glucose flux) but is up-regulated in cellular stress (30 % of glucose flux) [8]. Our study indicated that this up-regulation occurred in patients with high oxidative load, such as those with systemic inflammation (all our patients fulfilled diagnostic criteria for SIRS). This finding is consistent with the current literature, which suggests critical illness generated a high oxidative burden in systemic circulation, especially in sepsis and SIRS patients [11, 17]. We also found that, in critically ill patients, this up-regulation was accompanied by a reduction in the biosynthetic part of the pentose phosphate pathway. This suggested that the cells had reprioritized its metabolic need towards redox homeostasis (to repair injury), rather than nucleic acid synthesis (to support growth). Additionally, we found evidence of inhibition in the electron transport chain (the main producer of ROS under normal condition), indicating that the cell may have entered into a self-protective mode by reducing pro-oxidants production. Collectively, these findings represent cellular shifts towards increasing NADPH while simultaneously decreasing oxidative load. Such shifts may help cells minimize oxidative damage caused by inflammatory cytokines in blood.

Our study showed that an increased dependence on glycolysis could indeed occur in aerobic conditions. The traditional paradigm predicts that, in aerobic conditions, mitochondrial oxidation is the preferred metabolic pathway [3]. Our finding showed an alternative scenario. Such a finding suggested that the traditionally held assumption that increased glycolysis (and hence hyperlactataemia) is an evidence of hypoxia or ischemia may have limitations. It is likely that an increased aerobic glycolysis may represent an adaptive physiological response to sepsis or systemic inflammation.

It is important to note that our findings are in keeping with recent advances on the study of cellular metabolism. First, recent studies have found that metabolic reprogramming has occurred in human leukocytes. In the work performed by Liu et al., human leukocytes (monocytes) underwent metabolic reprogramming in the early phase of sepsis [18]. In the work performed by Tannahill et al., macrophages stimulated by lipopolysaccharide underwent up-regulation of glycolysis [19]. Our study showed that metabolic reprogramming has occurred in whole blood. Second, there is extensive evidence that demonstrates the existence of each component of the metabolic reprogramming, including aerobic glycolysis, pentose phosphate pathway up-regulation and tricarboxylic acid cycle down-regulation [16]. We noted that all of these components were evident in our findings. Third, there is evidence to show that cellular stress (e.g. radiation injury, trauma) or adrenergic stimulation (e.g. shock) triggers metabolic programming in the injured cells [20–23]. Our findings confirmed this observation and further extended it by demonstrating its presence in an intensive care setting. Put together, our study adds substantial new evidence to support the notion that cellular metabolism may play an important role in regulating host response to critical illness.

This current study differs from previous ones in the following aspects. First, we identified significant global changes across multiple cellular pathways. In contrast, previous studies focused on individual genes and their specific role in cellular metabolism. The global approach adopted in this study revealed important new findings that would otherwise been impossible to obtain in single gene studies. Second, our target tissue was circulating leukocytes, the pivotal cells that mediate host immune response to critical illness. Important physiological changes occurring in these cells could impact on disease outcomes and may provide new targets for novel drug therapy. Third, our findings were observed in a wide variety of medical/surgical conditions and in patients with different disease severity. This indicates that our findings were robust. Furthermore, it suggests that metabolic reprogramming may be a well-conserved response in a range of critical illnesses.

There are several limitations in our study. First, our findings were derived from transcriptomic analysis; our study did not provide data from metabolic assays or directly measure the degree of oxidative stress inside the cells. However, this had been previously demonstrated by Tannahill et al. [19]. Nevertheless, it would be useful to collaborate gene-expression data with metabolic/redox measurements, and indeed, we plan to perform such a study in the future. Second, the increased expression levels of certain genes may be confounded by variations in cell count within leukocyte subsets (e.g. increased neutrophils in sepsis/SIRS). We addressed this issue in a post hoc analysis; this analysis did not find any correlation between neutrophils counts and glycolytic genes (Additional file 3). Such a finding is in keeping with a previous study showing that an increase in glycolytic enzyme activity was independent of neutrophil counts [22]. Nevertheless, we could not exclude the effect of cell count on gene-expression (such effect, if present, was likely to be very small). Third, our samples were whole blood, which consisted of heterogeneous populations of different leukocyte subsets (e.g., lymphocytes, neutrophils and monocytes). We do not know whether metabolic reprogramming in different cell subsets occurred to a different extent. We plan to address this study limitation in future studies where cell subsets (instead of whole blood) will be used. Fourth, our cohort included severely ill patients. It is not known whether patients with mild to moderate illness also show similar findings. This is a further limitation of the study, and it warrants further investigation in the future.

Our findings have further research implications. Antioxidant therapy in critically ill patients has attracted much attention in recent years [24]. However, current antioxidant agents (e.g. selenium, glutamine) have not demonstrated success in improving patient outcomes in recent clinical trials [25]. New antioxidant agents are therefore urgently needed. Here, we provide a detailed roadmap of metabolic reprogramming in circulating leukocytes of critically ill patients. Further study of this road map and its main regulator gene may identify highly valuable drug targets for antioxidant therapy in critical illness.

## Conclusions

During critical illness, our study suggests that non-hypoxic cells up-regulate the glycolytic pathway to re-direct substrates towards NADPH synthesis and to maintain redox homeostasis. This previously under-recognized physiological phenomenon may reveal important new drug targets for future development of antioxidant therapy.

## Abbreviations

SIRS, systemic inflammatory response syndrome

## Additional files

Additional file 1: Full analysis results of metabolic pathways. Additional file 2: Analysis of regulatory genes that govern cell proliferation/growth. Additional file 3: Correlation between neutrophils counts and glycolytic genes.
